# Performance Analysis of Visible Light Communication Using CMOS Sensors

**DOI:** 10.3390/s16030309

**Published:** 2016-02-29

**Authors:** Trong-Hop Do, Myungsik Yoo

**Affiliations:** School of Electronic Engineering, Soongsil University, Seoul 06978, Korea; dotronghop@gmail.com

**Keywords:** visible light communication, image sensor, performance, analysis, CMOS

## Abstract

This paper elucidates the fundamentals of visible light communication systems that use the rolling shutter mechanism of CMOS sensors. All related information involving different subjects, such as photometry, camera operation, photography and image processing, are studied in tandem to explain the system. Then, the system performance is analyzed with respect to signal quality and data rate. To this end, a measure of signal quality, the signal to interference plus noise ratio (SINR), is formulated. Finally, a simulation is conducted to verify the analysis.

## 1. Introduction

There are two types of light receivers that can be used for visible light communication (VLC): photo diodes and image sensors. Photo diodes have been widely used thanks to their low cost and high reception bandwidth. Image sensors, on the other hand, are still far behind, since traditionally, they are more expensive, and the achievable data rate is lower. In recent years, image sensor technology has made a big leap with regard to price and performance. Even the least expensive smartphones nowadays are equipped with high-resolution CMOS sensor cameras. Many of them can shoot full HD videos at 30 or even 60 fps. This motivates the use of CMOS sensors for VLC.

As in any communication system, achieving a high data rate is one of the first targets. There are some approaches for achieving a high data rate using an image sensor. First, a high-speed camera can be used to receive the high-modulation-frequency light from an LED. The clear disadvantage of this approach is the cost of a high speed camera. Even though the megapixel war in the camera industry has pushed the resolutions of image sensors to very high levels and enabled high-resolution image sensors to be sold at low prices, it is still impossible to have a high-frame-rate camera at low cost. The second approach is using an LED array to transmit LED patterns, which conveys multiple bits per frame, at a low frequency to a normal camera. This approach has the limitation that the low frequency blinking of the LED can cause flickering that is perceived by human eyes. The third approach takes advantage of the rolling shutter mechanism of CMOS sensors to receive multiple bits modulated at a high frequency within one frame [1]. As a result, flickering is unobserved regardless of the low frame rate. There are special approaches, such as the one proposed in [2], which developed an optical communication image sensor capable of responding promptly to the variation of LED light. While that technique can achieve a very high data rate up to 20 Mbps, it requires a very proprietary sensor, which is not available for every researcher to use for designing a VLC system.

In [1], the concept of using the rolling shutter effect of CMOS sensors for VLC has been proposed. This technique has been reviewed in many studies [3,4,5,6,7]. Some have applied this technique for vehicular communication [8] and for positioning [9,10]. However, the current knowledge of this technique has many gaps. That is because the theoretical foundation necessary for understanding the operation of the system has not been presented in previous papers. More importantly, the system performance with regard to signal quality and data rate has never been analyzed, not to mention the lack of a formal measure of signal quality. Consequently, many questions related to the impacts on the system performance of parameters, such as sensor readout time, exposure time, LED modulation frequency and ambient light level, remained unanswered. For example, given am LED with a specific lumen output, it is unknown what the proper exposure time should be. Given a camera with a specific frame rate, there is a question of how high of a data rate can be achieved; or one might ask which setting should be changed to improve the signal quality.

In this paper, firstly, all background knowledge in various subjects, including photometry, camera operation, photography and image processing, is gathered, and the relations between them are clarified to explain the whole process of the system. Then, from that prerequisite knowledge, the system performance with respect to signal quality and data rate is analyzed, and thus, the questions raised above are answered. To analyze the signal quality, first, the two main factors affecting the recognizability of the input signal in an image (they are intersymbol interference and ambient light noise) are explained. Secondly, the measurement for signal quality is formally defined as the signal to interference plus noise ratio (SINR). Then, the effects of system parameters on SINR and data rate are analyzed, and the equations for calculating SINR and the data rate from the given parameters are formulated. Using these formulae, one can estimate the signal quality and data rate beforehand and, thus, can change the system parameters to obtain the desired performance. Finally, a simulation is conducted to verify the analysis.

## 2. Fundamental of the System

### 2.1. Operation of CMOS Sensors and Camera

#### 2.1.1. Rolling Shutter Mechanism and its Advantage in VLC

**Figure 1:**
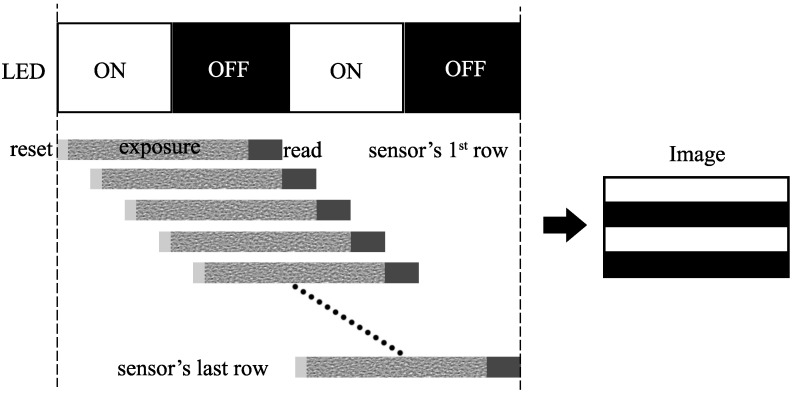
Image acquisition in a CMOS sensor.

Figure 1 illustrates image acquisition with a CMOS sensor. With a CCD sensor, the whole sensor surface is exposed at the same time, and the data in all pixels are read out at the same time. With a CMOS sensor, the exposure and data readout are performed row by row. The delay time between the exposures of two rows is equal to the readout time of one row. This mechanism is called the rolling shutter of the CMOS sensor. The time for the integration of one frame starts when the first row is reset and finishes when the last row is read out. During this period, turning on and off the LED light would result in light and dark bands on the image. By this mechanism, several bits, which are represented by these bands, can be received in a single frame.

#### 2.1.2. Frame Rate in a CMOS Sensor Camera

**Figure 2:**
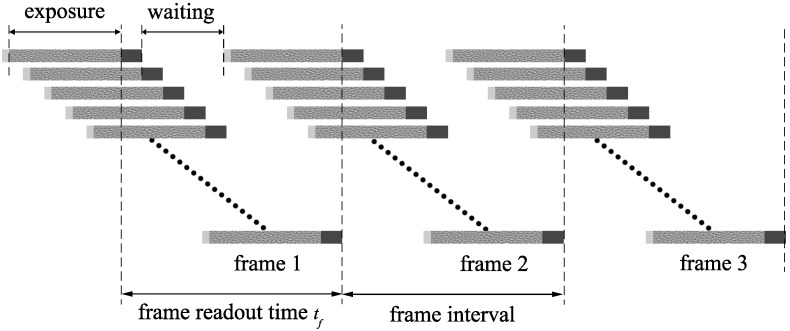
Image acquisition in multiple frames.

Basically, a row in a CMOS sensor is ready to be exposed for the next frame as soon as the readout in that row for the current frame has finished. Thus, the first row in the sensor might start its exposure for the next frame, while the last rows in the sensor are still being exposed for the current frame, as described in Figure 2. In other words, the start of the image acquisition for the next frame does not need to wait for the completion of the image acquisition for the current frame. This mechanism helps the CMOS sensor cameras achieve relatively higher frame rates than their CCD counterparts.

Obviously, the sooner the first row in the sensor starts its exposure for the next frame, the higher the frame rate that is achieved. However, at any specific time, the readout process can occur at only one row. Hence, to keep the exposure time the same between frames, the first row needs to wait for a period of time so that the readout for the next frame in that row occurs after the readout for the current frame in the last row has finished. Consequently, the minimum interval between frames is equal the frame readout time $t_{f}$, as described in Figure 2.

Corresponding to the minimum frame interval, the maximum frame rate is determined as:

(1) $$\begin{array}{c} FR_{max}=\frac{1}{t_{f}} \end{array}$$

While some cameras, such as the ones in the Nikon 1 series, can manage to achieve the above maximum frame rate, many other cameras can only offer the frame rate of 60% to 90% of the maximum frame rate [11]. This is because in their design, these cameras have been allowed a period, a “guard” period, which is used for the setting of the next frame, as well as other purposes. In these cases, the first row in the sensor needs to wait for a longer time before starting its exposure for the next frame. More specifically, the frame interval in these cases would equal the readout time plus the guard period. Therefore, in general, the frame rate of the camera is determined as:

(2) $$\begin{array}{c} FR=\frac{1}{t_{f}+t_{g}} \end{array}$$

where $t_{g}$ is the guard period between frames.

### 2.2. Calculating Pixel Value

#### 2.2.1. The Whole Process from Receiving Light to the Calculating Pixel Value in Image Sensor

**Figure 3:**
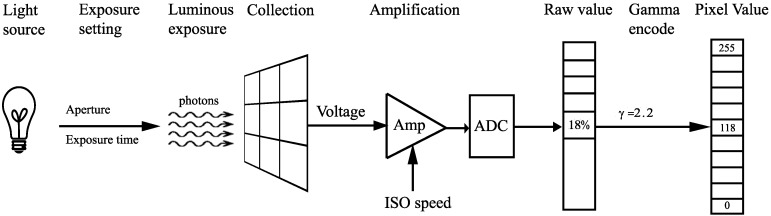
The whole process for calculating the pixel value.

The whole process for calculating a pixel value is described in Figure 3. The light goes through the lens and falls onto the image sensor. Depending on the exposure setting, which determines the lens aperture and exposure time in the camera, a specific luminous exposure, which represents the amount of light per unit area, will be received by the sensor. After that, the photon energy will be converted to a voltage. In the amplification process, a factor called International Standards Organization (ISO) speed will determine how much the voltage is amplified. Through the amplifier and analog-to-digital converter, this voltage will be represented by a digital number that is usually called the raw output (or raw pixel value) of the sensor. Finally, a gamma encoding operation will convert this raw output value to a pixel value.

In this paper, to analyze the signal quality and to simulate the system, the pixel value at given lighting conditions and camera settings needs to be calculated. In the process shown in Figure 3, the luminous exposure can be calculated accurately with given assumptions. Additionally, the pixel value can also be calculated from the raw output value if the value of gamma is given. However, the voltage generated at a given luminous exposure, as well as the raw output value representing a given voltage are really difficult to calculate, since these values are determined by many parameters, which cannot easily be assumed. Therefore, instead of strictly following all of the steps in Figure 3, this paper derives a shortcut method for calculating the pixel value directly from the light source and camera settings without going through the calculations of luminous exposure, output voltage and raw output values. This shortcut calculation will be the foundation for the analysis and simulation in this paper. The derivation of this shortcut calculation will be presented in the following sections.

#### 2.2.2. Calculating the Pixel Value from the Raw Output Value

The actual output voltage from each cell of an image sensor is directly proportional to the luminous exposure, and so is the raw output value. However, the response of human eyes to light is logarithmic in the sense that the eye response is proportional to the logarithm of the light intensity. Therefore, an operation called gamma encoding is applied to redistribute the raw outputs from the sensor to pixel values that are closer to how human eyes perceive them. Assume that an eight-bit RGB color space is used. The maximum pixel value is 255, and then, the gamma encoding is defined by the following power-law expression:

(3) $$\begin{array}{c} PV=255\times {\left(\frac{raw}{raw_{max}}\right)}^{1/\gamma } \end{array}$$

where PV is the pixel value, raw is the raw output value and $raw_{max}$ is the maximum possible raw output value, which is determined by the number of bits that the camera uses for representing the raw output value.

According to the standard [12], an object with actual luminous value (*i.e.*, the raw output value) of 18% of the full-scale would be rendered as middle gray, which is equivalent to 46% of the maximum digital brightness in the image. Therefore, the raw output value of 18% would be encoded into the pixel value of 118 with the full scale being 255. To accomplish this, the standard gamma value of 2.2 is used.

**Figure 4:**
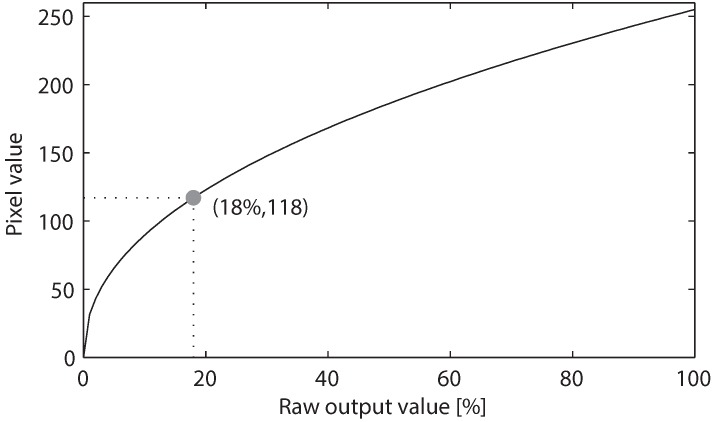
Gamma encoding of raw the pixel value with γ=2.2.

Figure 4 shows the gamma encoding operation, which translates a raw output data to a corresponding RGB pixel value with the value of gamma of γ=2.2. The middle gray point indicates the mapping of the raw output value of 18% of the full scale to the pixel value of 118. Note that 18%, 46%, 118 and 2.2 are rounded off values, which are conventionally presented in the literature. The actual value used by each camera manufacture for the gamma encoding might be slightly different. In this paper, the actual gamma used for the simulation is 2.22.

#### 2.2.3. Calculating the Pixel Value from the Luminous Exposure Ratio

Since the raw output value is directly proportional to the luminous exposure, it can be assumed that:

(4) $$\begin{array}{c} raw=Const\times H \end{array}$$

where *H* denotes the luminous exposure and Const is a specific constant.

Let $raw_{18\%}$ denote the raw output value equal to 18% of the full scale and $H_{SOS}$ denote the luminous exposure corresponding to the raw output of $raw_{18\%}$. This means:

(5) $$\begin{array}{c} raw_{18\%}=Const\times H_{SOS} \end{array}$$

Then,

(6) $$\begin{array}{c} \frac{raw}{raw_{18\%}}=\frac{H}{H_{SOS}} \end{array}$$

Since the raw output value of $raw_{18\%}$ is encoded to become the pixel value of 118 (*i.e.*, the middle gray point), from Equation (3), it can be seen that:

(7) $$\begin{array}{c} 118=255\times {\left(\frac{raw_{18\%}}{raw_{max}}\right)}^{1/\gamma } \end{array}$$

From Equations (3) and (7), the pixel value can be given as:

(8) $$\begin{array}{c} PV=118\times {\left(\frac{raw}{raw_{18\%}}\right)}^{1/\gamma } \end{array}$$

From Equations (6) and (8), the pixel value can be calculated from the luminous exposure ratio.

(9) $$\begin{array}{c} PV=118\times {\left(\frac{H}{H_{SOS}}\right)}^{1/\gamma } \end{array}$$

#### 2.2.4. Calculating Luminous Exposure Ratio from Exposure Difference

The luminous exposure is given by [13]:

(10) $$\begin{array}{c} H=\frac{qL_{v}t}{N^{2}} \end{array}$$

where $L_{v}$ is the luminance of the object (in cd/m${}^{2}$), *N* is the relative aperture (f-number) of the lens, *t* is the exposure time in seconds and *q* is a factor whose value is determined by the transmittance *T* of the lens, the vignetting factor v(θ) and the angle *θ* relative to the axis of the lens as:

(11) $$\begin{array}{c} q=\frac{\pi }{4}Tv\left(\theta \right)\cos^{4}\left(\theta \right) \end{array}$$

For convenience, an exposure value EV is used to represent a combination of lens aperture and exposure time as [13]:

(12) $$\begin{array}{c} EV=\log_{2}\frac{N^{2}}{t} \end{array}$$

As expressed in Equation (10), the luminous exposure is directly proportional to the ratio $\frac{t}{N^{2}}$. Therefore, for any object with fixed luminance, all exposure settings that have the same EV would make the sensor receive the same luminous exposure and, thus, make that object rendered to the same brightness in the image. Thus, given an object with specific luminance, there is a unique exposure value, the “indicated exposure value”, that is required for the camera to receive the luminous exposure $H_{SOS}$, which makes the object rendered as middle gray.

Given a light source with specific luminance, suppose that $EV_{set}$ is the exposure value set for the camera and $EV_{ind}$ is the indicated exposure value. The exposure difference between the indicated and the set exposures, denoted by ED, is defined as:

(13) $$\begin{array}{c} ED=EV_{ind}-EV_{set} \end{array}$$

From Equations (10) and (12), it can be seen that:

(14) $$\begin{array}{c} ED=\log_{2}\frac{H}{H_{SOS}} \end{array}$$

where *H* is the luminous exposure received with the set exposure value and $H_{SOS}$ is the exposure received with the indicated exposure value.

Thus,

(15) $$\begin{array}{c} \frac{H}{H_{SOS}}=2^{ED} \end{array}$$

From Equations (9) and (15), the pixel value can be calculated from the exposure difference as:

(16) $$\begin{array}{c} PV=118\times 2^{ED/\gamma } \end{array}$$

As can be seen from Figure 4, when the exposure exceeds the maximum value that can be represented, a phenomenon called clipping occurs. The clipped area of the image will appear as a uniform area of maximum brightness, which is 255 in the eight-bit RGB color space. Therefore, Equation (16) is valid only when the exposure difference is smaller than a specific value:

(17) $$\begin{array}{c} ED<\gamma \times \log_{2}\frac{256}{118}\approx 2.5 \end{array}$$

In Equation (13), while the set exposure value $EV_{set}$ can be easily calculated from the lens aperture and exposure time using Equation (12), the method for calculating the indicated exposure value $EV_{ind}$ is still unknown now. Therefore, in the following sections, the method for calculating $EV_{ind}$ from the given camera setting and light source intensity will be explained. First, the photometry of LED and ambient light is presented to explain how to calculate the intensity of the light source.

#### 2.2.5. Photometry of LED and Ambient Light

In the system considered in this paper, the subject of the image is the LED. Furthermore, it is assumed that the LED covers the whole image sensor. In other words, there are only two sources of light coming to the sensor: LED light radiated by the LED itself and ambient light bouncing off the LED surface.

##### Measurement of Radiated Light Intensity

The intensity of LED radiated light is measured in luminance, denoted as $L_{v}$, which indicates how much luminous power is perceived by the human eye looking at the surface from a particular angle [14]. The unit for luminance is candela per square meter (cd/m${}^{2}$). In practice, the luminance of the LED can be measured using a light meter or simply given by the LED manufacture.

##### Measurement of Incident Light Intensity

The intensity of the ambient light illuminating the LED surfaces is measured in illuminance, denoted as $E_{v}$, which indicates how much the incident light illuminates the surface [14]. The unit of illuminance is lux (lx). The illuminance of the ambient light can be measured by a light meter or given as an assumption. Usually, the illuminance at different environments is assumed to have the value given in Table 1.

Assume that the ambient light illuminance is $E_{v}$ and the reflectance of an object is *R*; the luminance of the reflected light from that object is given as [15]:

(18) $$\begin{array}{c} L_{v}=\frac{E_{v}\times R}{\pi } \end{array}$$

#### 2.2.6. Calculating Indicated Exposure Value from Given Camera Settings and Light Source Intensity

As explained earlier, given a specific luminous exposure, which corresponds to a specific output voltage, the ISO speed will determine the corresponding raw output value. Therefore, basically, any level of luminous exposure received by the sensor can be rendered as middle gray given an appropriate ISO speed. According to the standard output sensitivity (SOS) [12], the ISO speed that makes a certain luminous exposure value of *H* to be rendered as middle gray, by the camera manufacturer’s definition, has the value of *S* given as:

(19) $$\begin{array}{c} S=\frac{10}{H} \end{array}$$

Therefore, in any specific camera, given the ISO value set to *S*, the luminous exposure $H_{SOS}$ required for producing the middle gray tone in the image is given as:

(20) $$\begin{array}{c} H_{SOS}=\frac{10}{S} \end{array}$$

##### Indicated Exposure Value for Radiated Light

From Equations (10) and (20), the camera setting required for producing the middle gray tone for an object having the luminance $L_{v}$ is determined as:

(21) $$\begin{array}{c} \frac{N^{2}}{t}=\frac{L_{v}\times S}{K} \end{array}$$

where $K=\frac{10}{q}$ is the reflected light meter calibration constant.

From Equations (12) and (21), the indicated exposure value $EV_{LED}$ corresponding to the light radiated from the LED is given as:

(22) $$\begin{array}{c} EV_{LED}=\log_{2}\frac{L_{v}\times S}{K} \end{array}$$

where $L_{v}$ is the luminance in cd/m${}^{2}$ of the light radiated from the LED.

##### Indicated Exposure Value for Incident Light

From Equations (18) and (21), the required camera setting for producing the middle gray tone for an object illuminated by an ambient light having the illuminance of $E_{v}$ is given as:

(23) $$\begin{array}{c} \frac{N^{2}}{t}=\frac{E_{v}\times S}{C} \end{array}$$

where $C=\frac{10\pi }{qR}=K\frac{\pi }{R}$ is the incident light meter calibration constant.

From Equations (12) and (23), the indicated exposure value $EV_{BG}$ corresponding to the background ambient light bouncing off the LED surface is given as:

(24) $$\begin{array}{c} EV_{BG}=\log_{2}\frac{E_{v}\times S}{C} \end{array}$$

where $E_{v}$ is the illuminance in lux of the ambient light coming to the LED.

##### Indicated Exposure Value for the Combined Light Source

As mentioned earlier, the light coming from LED surface to the image sensor includes the light radiated by the LED and the ambient light bouncing off of the LED surface. Let $H_{LED}$, $H_{BG}$ and $H_{combine}$ respectively denote the luminous exposure received by the sensor from the LED radiated light, the reflected ambient light and the combined light sources. Let $EV_{set}$ denote the set exposure value.

From Equations (13) and (15), $H_{LED}$, $H_{BG}$ and $H_{combine}$ are given as:

(25) $$\begin{array}{c} \left\{\begin{array}{c} H_{LED}=H_{SOS}\times 2^{EV_{LED}-EV_{set}}\\ H_{BG}=H_{SOS}\times 2^{EV_{BG}-EV_{set}}\\ H_{combine}=H_{LED}+H_{BG}=H_{SOS}\times \frac{2^{EV_{LED}}+2^{EV_{BG}}}{2^{EV_{set}}} \end{array}\right. \end{array}$$

From Equation (14), the exposure difference is given as:

(26) $$\begin{array}{c} ED=\log_{2}\left(\frac{H_{combine}}{H_{SOS}}\right)=\log_{2}\left(2^{ED_{LED}}+2^{EV_{BG}}\right)-EV_{set} \end{array}$$

From Equation (13), the indicated exposure value corresponding to the combined light source is given as:

(27) $$\begin{array}{c} EV_{combine}=ED+EV_{set}=\log_{2}\left(2^{ED_{LED}}+2^{EV_{BG}}\right) \end{array}$$

From the indicated exposure value calculated above and the set exposure value $EV_{set}$ calculated using Equation (12), the exposure difference ED is calculated using Equation (13), and finally, the pixel value PV can be obtained using Equation (16).

## 3. Performance Analysis of the System

The performance of the VLC system using the CMOS rolling shutter mechanism will be analyzed with respect to two major aspects: signal quality and data rate. It is important to note that the analysis in this section is valid when the condition described in Equation (17) is satisfied. In other words, the prerequisite of the analysis is that the image is exposed correctly so that the highlight, the white band, is not clipped. This is a fair assumption, since the exposure time, as can be seen later in this section, should be kept as short as possible, and thus, clipping would never be the case in practice.

### 3.1. Signal Quality

The signal quality of VLC using CMOS sensors is considered high when the white and black bands, which express the logical one and zero, can be distinguished easily. In image processing, the distinguishability of these two bands is determined by the clarity and contrast of the image. While clarity expresses the local difference between white and black bands in transition regions of the image, contrast indicates the global difference between the maximum and minimum brightness of the entire image. Clarity can be considered to be the measurement for the intersymbol interference (ISI) effect, and contrast can be considered to be the measurement for the effect of ambient light noise. Therefore, similar to other wireless communication technology, the signal quality of VLC using CMOS sensors can also be measured by the SINR.

#### 3.1.1. Intersymbol Interference

**Figure 5:**
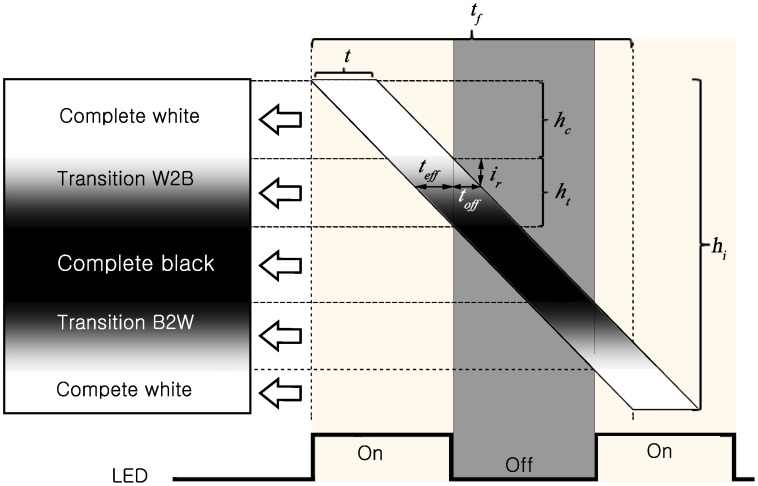
Transition between white and black bands.

Usually, the image clarity is reduced due to the presence of transitions between white and black bands. As the exposure time is longer than zero, there must be some rows at which the switching of the LED occurs during their exposure, and thus, the presence of transition bands is inevitable, as illustrated in Figure 5. In this figure: $t_{f}$ is the frame readout time; $h_{i}$ is the image height (*i.e.*, the number of rows in the sensor); $h_{t}$ is the height (*i.e.*, the number of rows) of the transition band; $h_{c}$ is the height (*i.e.*, the number of rows) of the complete band; *t* is the exposure time of the camera; and $t_{eff}$ and $t_{off}$ are respectively the effective and off exposure time of a row of the sensor.

As can be seen in Figure 5, the exposure time *t* is the time period that the sensor opens to receive light. However, if the LED switches during the exposure of a row, the exposure period of that row can be divided into two periods: effective exposure and off exposure. In the first period, the LED is on, and thus, the row received both LED light and ambient light. In the second period, the LED is off, and thus, the row receives only ambient light.

For each row in the transition band, let $i_{r}$ denote the relative position of that row with respect to the first row of the transition band. In other words, $i_{r}$ is the total number of rows counted from the first row in the corresponding transition band to that row. Then, the relative positions of rows, from the first to the final one, in the transition band are $i_{r}=1,2,3,...,h_{t}$.

From the property of similar triangles in Figure 5, the off exposure time of a row $i_{r}$ in a transition band is given as:

(28) $$\begin{array}{c} t_{off}=t_{f}\times \frac{i_{r}}{h_{i}}=i_{r}\times t_{r} \end{array}$$

where $t_{r}$ is the row readout time given as:

(29) $$\begin{array}{c} t_{r}=\frac{t_{f}}{h_{i}} \end{array}$$

Then, the effective exposure time of a row $i_{r}$ in a transition band is given as:

(30) $$\begin{array}{c} t_{eff}=t-i_{r}\times t_{r} \end{array}$$

Furthermore, from the property of similar triangles in Figure 5, the height of the transition band is given as:

(31) $$\begin{array}{c} h_{t}=i_{r}\times \frac{t}{t_{off}}=\frac{t}{t_{r}} \end{array}$$

The height of the complete white (or black) band in the number of rows $h_{c}$ in Figure 5 is given as:

(32) $$\begin{array}{c} h_{c}=\frac{1/f_{led}-t}{t_{r}}=\frac{1}{f_{led}\times t_{r}}-h_{t} \end{array}$$

where $f_{led}$ is the blinking frequency of the LED.

It is obvious that the heights of the transition bands should be as small as possible. The row readout time is a specification that cannot be changed for a specific camera. However, the transition between white and black bands, as shown in Equation (31), can be reduced by shortening the exposure time.

In [6], the authors conducted experiments with various LED frequencies and observed that when the LED frequency was higher than the reciprocal of the exposure time, the camera was not able to record the signal. While the reason for this phenomenon was not covered in [6], it can be seen clearly through Equation (32). From this equation, it turns out that the complete band has zero height when the exposure time equals the LED duration. Therefore, the exposure time must be shorter than the LED pulse duration:

(33) $$\begin{array}{c} t<1/f_{led} \end{array}$$

**Figure 6:**
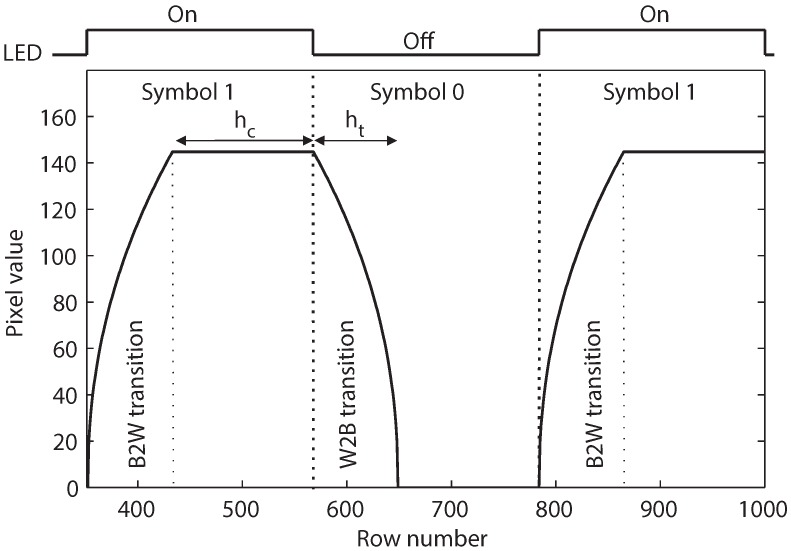
The intersymbol interference (ISI) caused by the transition band.

Figure 6 explains why the transition bands in the image correspond to the ISI of the signal. In this figure, the pixel value can be considered to be the signal amplitude, and thus, the white and black bands in the image correspond to pulses that represent the symbols 1 and 0. The black-to-white transition corresponds to the interference that symbol 0 introduces to symbol 1. In contrast, the white-to-black transition corresponds to the interference that symbol 1 introduces to symbol 0. These ISIs appear at the beginning of each symbol, causing the slow rising of symbol 1 and the slow falling of symbol 0, as well as reducing the distinguishability of the two symbols from each other.

#### 3.1.2. Ambient Light Noise

In the indoor environment, the luminance of the LED is much higher than the ambient light. For example, the indicated exposure value at ISO 100 of a typical LED is 17, whereas that of a typical working office is seven. This means the LED is $2^{10}$-times brighter than the ambient light. When the camera exposure is set for the LED, the ambient light in this case has almost no impact on the image.

**Figure 7:**
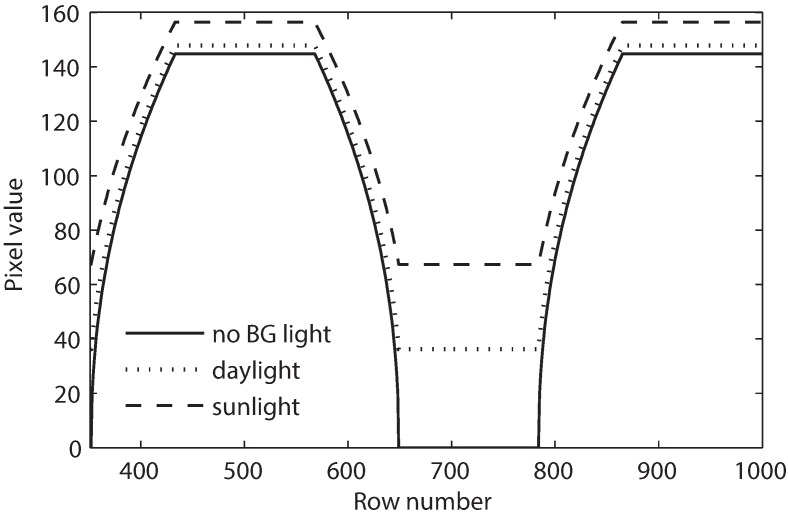
Effect of ambient light.

However, in an outdoor environment, the ambient light can bring both the highlight and shadow of the image up. However, because of the non-linear response between the exposure and pixel value, the ambient light shows a much stronger impact on the shadow than on the highlight. In other words, the ambient light significantly raises the pixel level of the logical zero (black) while it brings about a small increase in the pixel level of the logical one (white), as shown in Figure 7. Therefore, in an outdoor environment, the ambient light can considerably reduce the image contrast. To analyze the effect of ambient light, the minimum and maximum pixel values need to be calculated and compared.

The rows that fully receive the LED light and ambient light during their exposure period would have the maximum pixel value in the image. The maximum pixel value is given as (Appendix A):

(34) $$\begin{array}{c} PV_{max}=118{\left(\frac{S\times t}{K\times N^{2}}\left(L_{v}+E_{v}\frac{R}{\pi }\right)\right)}^{1/\gamma } \end{array}$$

The rows that do not receive any LED light during their exposure period would have the minimum pixel value in the image. Since ambient light is the only light source, the exposure difference would be the difference between the indicated EV of ambient light and the set exposure. Then, the minimum pixel value is given as (Appendix B):

(35) $$\begin{array}{c} PV_{min}=118{\left(\frac{S\times t}{K\times N^{2}}\times E_{v}\frac{R}{\pi }\right)}^{1/\gamma } \end{array}$$

Note that in the two equations above, $E_{v}\frac{R}{\pi }$ is the luminance caused by the ambient light bouncing off the LED surface.

The contrast between the white and black bands is given by:

(36) $$\begin{array}{c} Contrast=PV_{max}-PV_{min}=118\left({\left(L_{v}+E_{v}\frac{R}{\pi }\right)}^{1/\gamma }-{\left(E_{v}\frac{R}{\pi }\right)}^{1/\gamma }\right){\left(\frac{S\times t}{K\times N^{2}}\right)}^{1/\gamma } \end{array}$$

The derivative of the contrast with respect to $E_{v}$ is given by:

(37) $$\begin{array}{c} \frac{\partial Contrast}{\partial E_{v}}=\frac{R}{\pi g}\left({\left(L_{v}+\frac{R}{\pi }E_{v}\right)}^{1/\gamma -1}-{\left(\frac{R}{\pi }E_{v}\right)}^{1/\gamma -1}\right) \end{array}$$

Since 1/γ−1<0 and $L_{v}+E_{v}\frac{R}{\pi }>E_{v}\frac{R}{\pi }$, $\frac{\partial Contrast}{\partial E_{v}}<0$. Thus, the contrast decreases when the ambient light increases. This explains the significant increasing of the pixel level of the logical zero compared to that of the logical one when the ambient light increases, as represented in Figure 7.

#### 3.1.3. SINR

**Figure 8:**
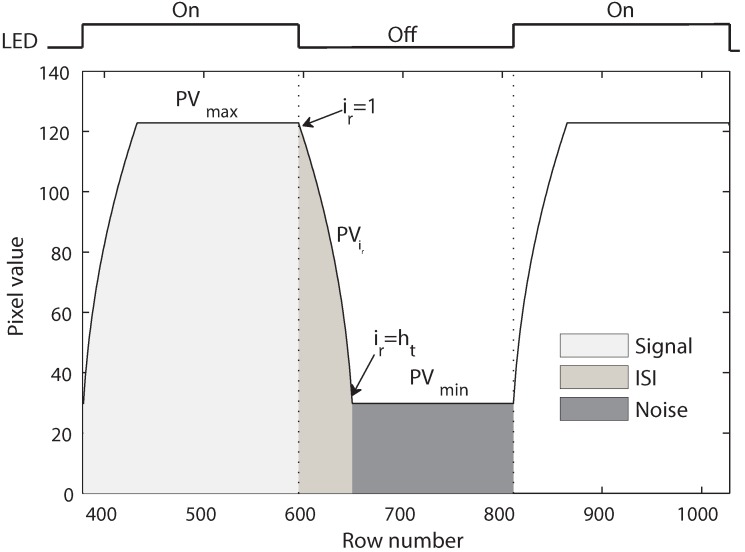
Signal to interference plus noise ratio.

A typical signal with the presence of ISI and ambient noise is illustrated in Figure 8. $PV_{max}$ and $PV_{min}$ are the maximum and minimum pixel values in the image. $PV_{i_{r}}$ denotes the pixel value at a row having the relative position $i_{r}$ in the transition band (*i.e.*, the ISI part).

The signal to interference plus noise ratio (SINR) is given as:

(38) $$\begin{array}{c} SINR=\frac{Signal}{ISI+Noise} \end{array}$$

Since the ISI at the beginning of symbol 1 and the ISI at the beginning of symbol 0 are symmetric, Equation (38) can be expressed as:

(39) $$\begin{array}{c} SINR=\frac{\sum_{i_{r}=1}^{h_{t}}PV_{i_{r}}+h_{c}\times PV_{max}}{\sum_{i_{r}=1}^{h_{t}}PV_{i_{r}}+h_{c}\times PV_{min}} \end{array}$$

The values of $PV_{max}$ and $PV_{min}$ are given by Equations (34) and (35), respectively. The value of $PV_{i_{r}}$ is given by (Appendix C):

(40) $$\begin{array}{c} PV_{i_{r}}=118{\left(\frac{S\times t}{K\times N^{2}}\left(L_{v}\frac{h_{t}-i_{r}}{h_{t}}+E_{v}\frac{R}{\pi }\right)\right)}^{1/\gamma } \end{array}$$

From Equations (34), (35), (39) and (40), the value of SINR is given as:

(41) $$\begin{array}{c} SINR=\frac{{\sum_{i_{r}=1}^{h_{t}}\left(L_{v}\frac{h_{t}-i_{r}}{h_{t}}+E_{v}\frac{R}{\pi }\right)}^{1/\gamma }+h_{c}{\left(L_{v}+E_{v}\frac{R}{\pi }\right)}^{1/\gamma }}{{\sum_{i_{r}=1}^{h_{t}}\left(L_{v}\frac{h_{t}-i_{r}}{h_{t}}+E_{v}\frac{R}{\pi }\right)}^{1/\gamma }+h_{c}{\left(E_{v}\frac{R}{\pi }\right)}^{1/\gamma }} \end{array}$$

To examine the effects of system parameters on SINR, the differentials of SINR to related parameters in Equation (41) are calculated. For the sake of simplicity of calculation, firstly, the finite sum in Equation (41) is approximated by the integral:

(42) $$\begin{array}{c} \begin{array}{c} \sum_{i=1}^{h_{t}}{\left(L_{v}\frac{h_{t}-i_{r}}{h_{t}}+E_{v}\frac{R}{\pi }\right)}^{1/\gamma }\approx \int_{0}^{h_{t}}{\left(L_{v}\frac{h_{t}-i_{r}}{h_{t}}+E_{v}\frac{R}{\pi }\right)}^{1/\gamma }di_{r}=\frac{h_{t}\left({\left(L_{v}+E_{v}\frac{R}{\pi }\right)}^{1/\gamma +1}-{\left(E_{v}\frac{R}{\pi }\right)}^{1/\gamma +1}\right)}{L_{v}\left(1/\gamma +1\right)} \end{array} \end{array}$$

From Equations (41) and (42), the value of SINR is approximated as:

(43) $$\begin{array}{c} SINR\approx \frac{\frac{t\left({\left(L_{v}+E_{v}\frac{R}{\pi }\right)}^{1/\gamma +1}-{\left(E_{v}\frac{R}{\pi }\right)}^{1/\gamma +1}\right)}{L_{v}\left(1/\gamma +1\right)}+\frac{1-t\times f_{led}}{f_{led}}{\left(L_{v}+E_{v}\frac{R}{\pi }\right)}^{1/\gamma }}{\frac{t\left({\left(L_{v}+E_{v}\frac{R}{\pi }\right)}^{1/\gamma +1}-{\left(E_{v}\frac{R}{\pi }\right)}^{1/\gamma +1}\right)}{L_{v}\left(1/\gamma +1\right)}+\frac{1-t\times f_{led}}{f_{led}}{\left(E_{v}\frac{R}{\pi }\right)}^{1/\gamma }} \end{array}$$

It can be seen through Equation (43) that the parameter $t_{r}$ can be canceled out from the equation for SINR. Therefore, SINR is independent of the frame readout time.

The results (Appendix D, Appendix E and Appendix F) show that:

(44) $$\begin{array}{c} \left\{\begin{array}{c} \frac{\partial SINR}{\partial t}<0\\ \frac{\partial SINR}{\partial f_{led}}<0\\ \frac{\partial SINR}{\partial E_{v}}<0\\ \frac{\partial SINR}{\partial L_{v}}=0\;\text{when}\;E_{v}=0,\\ \frac{\partial SINR}{\partial L_{v}}>0\;\text{when}\;E_{v}>0 \end{array}\right. \end{array}$$

Therefore, SINR decreases when either the exposure time *t*, or the LED frequency $f_{led}$, or the ambient light illuminance $E_{v}$ increases. The effect of LED luminance depends on the ambient light. Without the presence of ambient light, the SINR is independent of the LED luminance. In contrast, with the presence of ambient light, the SINR increases when the LED luminance increases.

### 3.2. Data Rate

From Figure 5, we see that the total number of complete white and black bands per frame $N_{b}$ can be calculated as:

(45) $$\begin{array}{c} N_{bpf}=\frac{h_{im}}{h_{c}+h_{t}}=h_{im}\times t_{r}\times f_{led}=t_{f}\times f_{led} \end{array}$$

From Equations (2) and (45), the data rate is given by:

(46) $$\begin{array}{c} DR=FR\times N_{bpf}=\frac{t_{f}\times f_{led}}{t_{f}+t_{g}} \end{array}$$

It is easy to see that increasing the LED frequency will increase the data rate. However, there is a maximum value that the frequency of modulation of the LED cannot exceed. This maximum LED frequency can be determined through Equation (33) as $f_{led}<1/t$.

When $t_{guard}=0$, the camera has the maximum frame rate, and the data rate at that time is equal to the LED frequency. Additionally, because of Equation (33), the maximum data rate that can be achieved is:

(47) $$\begin{array}{c} DR_{max}=\frac{1}{t} \end{array}$$

### 3.3. Required Distance from LED to Camera

The major drawback of the technique using the rolling shutter CMOS sensor for VLC is that it require a close distance from the LED to the camera, so that the LED covers the entire or a big part of the sensor. For example, [1] and this paper assume that the LED covers the whole sensor. In [6], the LED only covers the vertical part of the sensor.

**Figure 9:**
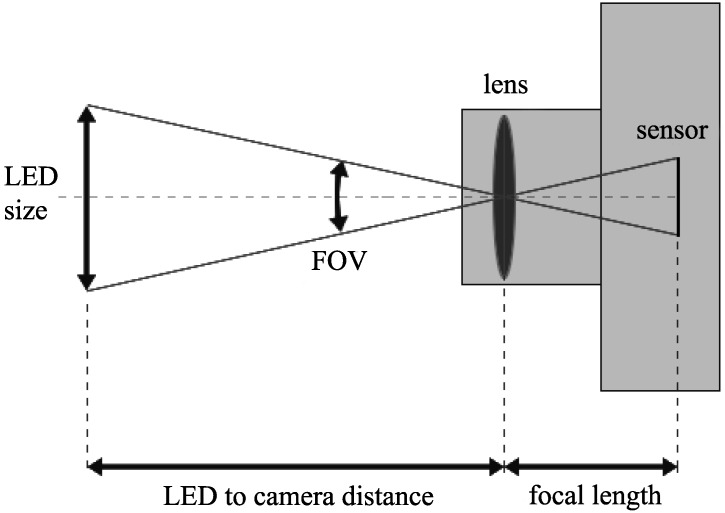
Relation between LED size, camera FOV and distance from the LED to the camera.

As shown in Figure 9, the required distance from the LED to the camera is determined by the LED size and the field of view (FOV) of the lens. A lens in a typical smartphone camera has a field of view equivalent to a focal length of a 35-mm lens on a full-frame camera. More specifically, a typical smartphone camera lens has a field of view of $54.4^{\circ }$ horizontally and $37.8^{\circ }$ vertically.

From Figure 9, the required distance from the LED to the camera is given by:

(48) $$\begin{array}{c} distance=\frac{LED\_size/2}{tan(FOV/2)} \end{array}$$

where LED_size is the diameter of the LED and FOV is the field of view of the lens.

For the LED to cover the whole sensor as in [1] and this paper, the horizontal part of the sensor must be covered within the FOV. Thus, given that the LED diameter is 10 cm and the lens has a field of view equivalent to a focal length of a 35-mm lens of a full-frame camera, the required distance from the LED to the camera should be 9.7 cm. For the LED to cover just the vertical part of the sensor as in [6], the vertical part of the sensor must be covered within the FOV. In this case, the required distance from the LED to the camera should be 14.6 cm. A longer required distance from the LED to the camera can be obtained by using a longer lens or increasing the size of the LED.

## 4. Simulation

### 4.1. Simulation Environment

To verify the analysis, we conducted a simulation of the system using MATLAB. The simulation reproduces the operation of a CMOS sensor as described in Section 2 to create a gray scale image. The LED luminance is assumed to have three levels, 4096 cd/m${}^{2}$, 8192 cd/m${}^{2}$ and 16,384 cd/m${}^{2}$, which correspond to the indicated exposure values of 15, 16 and 17, respectively.

ISO 2720:1974 [16] recommends that the ranges for the reflected-light meter calibration constant *K* be from 10.6–13.4. This paper assumes the usual value K=12.5, which is used by Canon, Nikon and Sekonic. The incident-light meter calibration constant *C* is determined based on the reflectance of the LED surface. Following [17], the reflectance of the transparent surfaces was assumed to be 40%. Then, the value of *C* is assumed to be $12.5\times \frac{\pi }{0.4}=98$.

It is assumed that the LED has the typical modulation bandwidth of 10 MHz or above. Given that the maximum modulation frequency of the LED is 8000 Hz, the slow rising and falling of the LED would cause very little effect on the ISI. For simplicity, in the simulation, the LED is assumed to switch instantly between on and off.

All of the simulation parameters are listed in Table 2.

### 4.2. Simulation and Calculation Procedure

**Figure 10:**
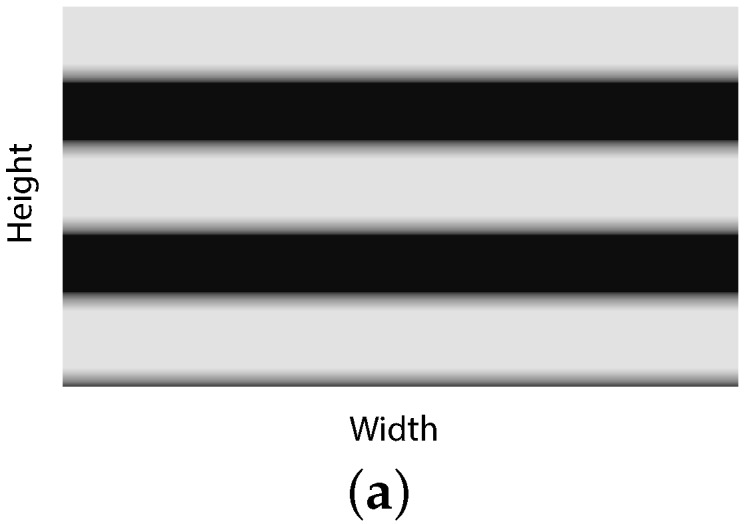

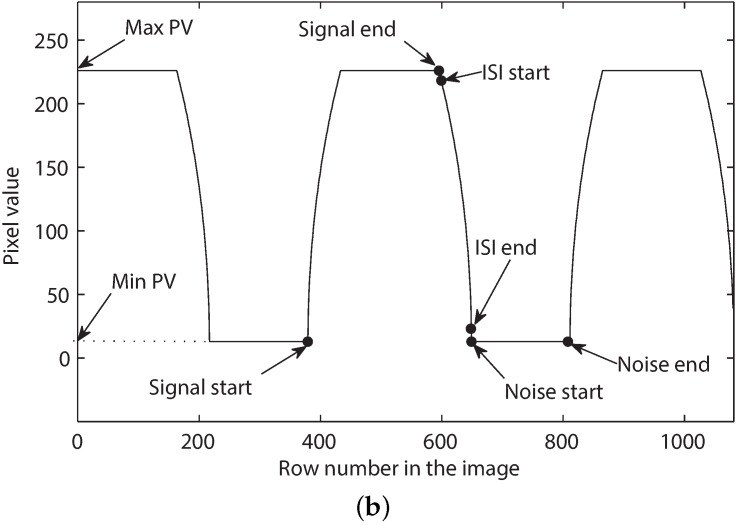
Simulated image and method for calculating SINR. (**a**) Simulated image; (**b**) Pixel value in each row of the simulated image.

Given a set of parameters with specific values, the pixel value of each row is calculated using the equations in Section 2, and an image is created as shown in Figure 10a. After that, the pixel value of each row is tracked to find the signal, ISI and noise parts in the image. As illustrated in Figure 10b, first, the maximum and minimum pixel values of the image are found. Then, the signal component starts where the pixel value increases from the minimum and ends where the pixel value decreases from the maximum. The ISI component begins right behind the end of the signal part and completes where the pixel value reaches the minimum. The noise is determined as the part having the minimum pixel value after the ISI. The signal, ISI and noise are calculated as the summation of the pixel value from the starting row to the ending row of these components. Afterwards, the SINR is calculated using Equation (38).

### 4.3. Simulation Results

#### 4.3.1. SINR

**Figure 11:**
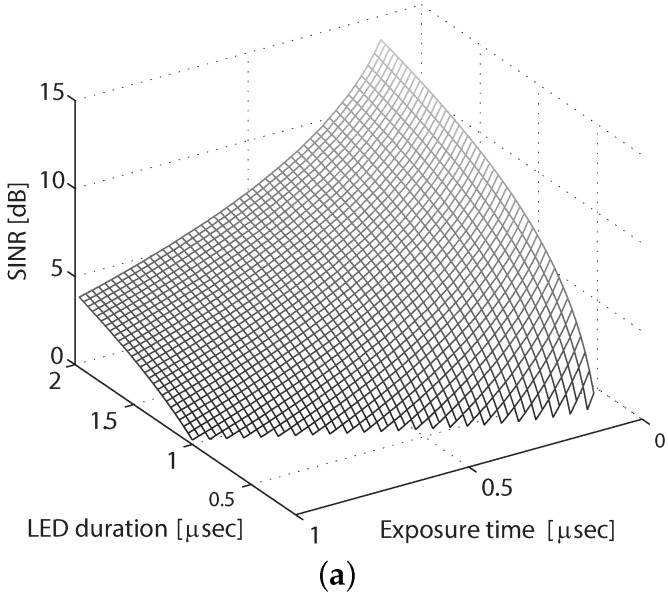

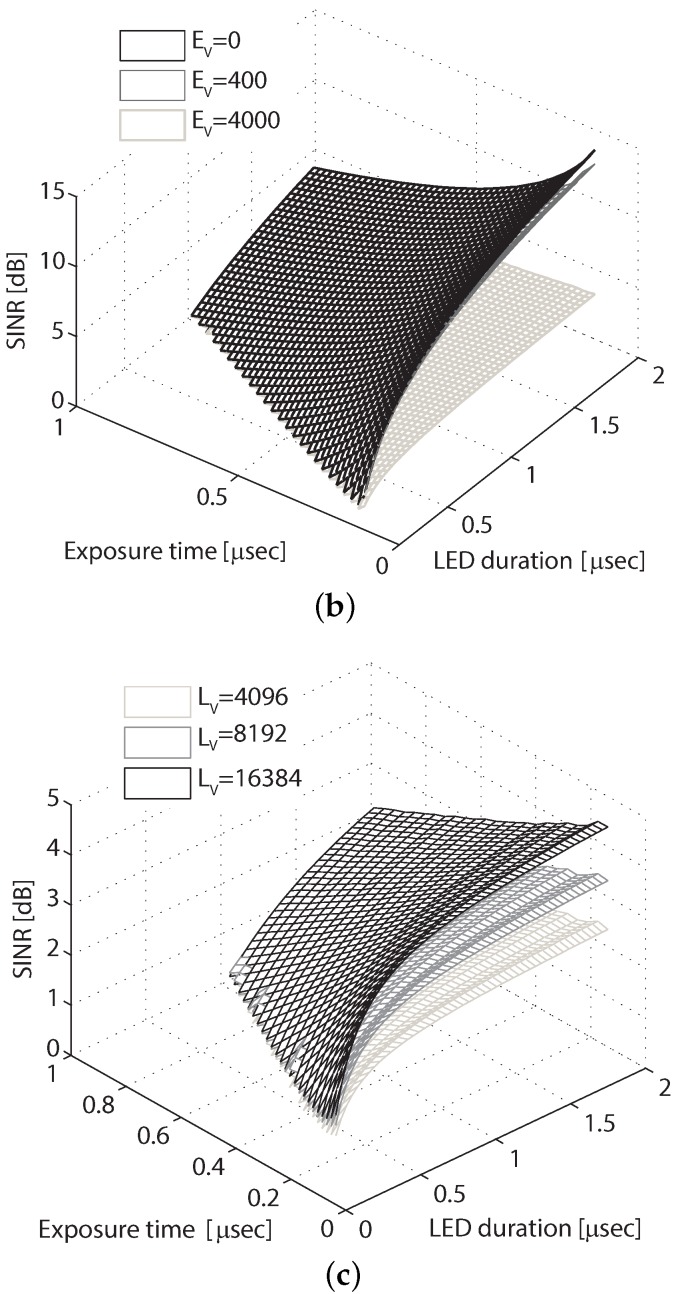
Effect of system parameters on the signal to interference plus noise ratio (SINR). (**a**) SINR with $E_{v}=0$, $L_{v}=8192$, $t_{f}=\frac{1}{100}$, $\frac{1}{8000}\leq t\leq \frac{1}{1000}$, $500\leq f_{led}\leq 8000$; (**b**) SINR with $L_{v}=8192$, $t_{f}=\frac{1}{100}$, $\frac{1}{8000}\leq t\leq \frac{1}{1000}$, $500\leq f_{led}\leq 8000$; (**c**) SINR with $E_{v}=$ 40,000, $t_{f}=\frac{1}{100}$, $\frac{1}{8000}\leq t\leq \frac{1}{1000}$, $500\leq f_{led}\leq 8000$.

According to the analysis, there are four parameters affecting SINR: exposure time, LED frequency, ambient light illuminance and LED luminance. Among them, the ambient light illuminance is usually given at certain environments and, thus, cannot be changed. The LED luminance is determined by the hardware and cannot be altered after the setting up of the system. However, the exposure time and LED frequency are two flexible parameters that can be changed to increase or decrease the system performance. Therefore, the simulation will focus on examining the effects of exposure time and LED pulse duration on the SINR. Nevertheless, the effects of ambient light illuminance and LED luminance are also tested. The simulation results are shown in Figure 11.

The simulation shown in Figure 11a uses fixed values for ambient light illuminance and LED luminance. More specifically, no ambient light and the LED luminance of 8192 lux are assumed. The exposure time ranges from 1/8000 s–1/1000 s, and the LED frequency ranges from 500 Hz–8000 Hz. Note that the LED duration is the reciprocal of the LED frequency, and in the simulation, the condition of $1/f_{led}>t$ is always guaranteed. The results show that SINR decreases when either exposure time or the LED frequency increases.

Figure 11b illustrates the effect of ambient light illuminance on SINR. Three kinds of environments are assumed: no ambient light, indoor office with 400 lux of illuminance and outdoor hazy sunlight with 40,000 lux of illuminance. It can be seen that SINR decreases when the ambient light illuminance increases. In addition, the ambient light in the indoor environment only has a subtle effect, as previously mentioned.

The LED luminance has no effect on SINR unless the ambient light exists. As previously shown, the presence of ambient light makes the SINR decrease. To lessen this undesired effect, the LED luminance should be increased. In the simulation shown in Figure 11c, an outdoor environment with strong ambient light of 40,000 lux is assumed. It can be seen that the SINR increases when the LED luminance increases.

As shown in the simulation, both shortening the exposure time and lengthening the LED duration increase the SINR. Lengthening the LED duration, however, would result in the decrease of the data rate. Consequently, the exposure time is the main parameter that can be changed to increase the SINR. Since reducing the exposure time makes the whole image darker, the ISO speed might need to be increased to compensate for the decrease of exposure. Note that increasing the ISO would introduce some noise in the image. The level of noise increase depends on the physical capability of the sensor. Therefore, when designing a VLC system using CMOS sensor, one needs to consider the effects of all of these parameters to get the most preferred performance.

#### 4.3.2. Data Rate

The data rate is mainly determined by the LED frequency, and the relationship between them is straightforward. For example, in Equation (46), if the guard time $t_{g}$ is assumed to be zero, then the data rate will be simply identical to the LED frequency. Therefore, a simulation showing the effect of the LED frequency on the data rate is not conducted in this paper. Instead, we just explain the maximum data rate that can be achieved in normal cases and simulate the frame captured at such a data rate.

As explained earlier, the LED frequency is limited by the exposure time and so is the data rate. In most prosumer cameras, whether mechanical shutters or electronic shutters are used, the minimum exposure time is set to 1/8000th of a second. They are made that way since the exposure time hardly needs to be smaller than 1/8000 s in normal applications. Using these cameras, the maximum data rate that can be achieved is 8 kbps.

In fact, when using an electronic shutter, the exposure time of the camera can be as short as the time required for switching the status of each row in the sensor from exposure to readout. For example, the exposure time on the Panasonic GH and Nikon 1 series can be set at 1/16,000 s, while that on the Fuji RX series is 1/32,000 s. Some scientific cameras even allow the exposure time to be set at a few microseconds. In a VLC system, an exposure time of a few microseconds would be impractical, since the light received by the sensor would be insufficient. Even the exposure time of 1/32,000 s would place strict requirements on the LED, lens and ISO setting. More specifically, to receive sufficient light at 1/32,000 s, the LED luminance should be high; the lens should have a large aperture; and the ISO speed should be set high.

**Figure 12:**
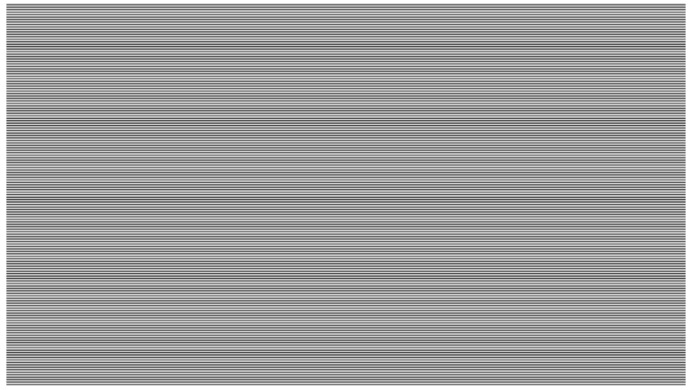
Simulated frame: t= 1/32,000, *f*-number =2.8, $L_{v}=16,384$, ISO=800.

Figure 12 shows a simulated frame corresponding to the setting: t=1/32,000, *f*-number =2.8, $L_{v}=16384$, ISO=800. The frame readout time is assumed to be 10 ms. Then, there are 320 bands in each frame. If the guard time between frames equals zero, the data rate would be 32 kbps. In normal cases, the lens aperture, LED luminance and ISO in this configuration almost reach their limits. For example, with prosumer cameras, an ISO higher than 800 would introduce too much noise in the image, and the lens aperture should be smaller than f/2.8. Therefore, a data rate of tens of kbps would be the maximum data rate of this technique in practical circumstances. Note that in the experiment conducted in [1], the maximum achieved data rate is 3.1 kbps.

## 5. Conclusions

Recently, visible light communication using rolling shutter CMOS sensors has been studied, and the results showed that it is a promising technique. In this study, the fundamentals of the system are explained in detail. After that, the system performance is analyzed with regard to signal quality and data rate. To accomplish this, a new measurement for signal quality is formally defined as a signal to interference plus noise ratio. Then, equations for calculating the SINR and data rate are formulated. Based on these equations, the effects of system parameters on the SINR and data rate are examined. Finally, a simulation is conducted to verify the analysis.

## Figures and Tables

**Table 1:** Illuminance at different environments.

Value (lux)	Environment
$10^{-4}$	Total starlight
1	Full moon
80	Hallways in office buildings
100	Very dark overcast day
300–500	Office lighting
400	Sunrise or sunset
1000	Overcast day
10,000–25,000	Full daylight
32,000–130,000	Direct sunlight

**Table 2:** Simulation environment.

Parameter	Value
Modulation	OOK
LED luminous intensity	0.73 (cd)
LED area	10 (cm${}^{2}$)
Indoor office illuminance	400 (lux)
Outdoor illuminance	40,000 (lux)
LED luminance	4096 to 16,384 (cd/m${}^{2}$)
ISO speed	100
Lens aperture	4
*γ*	2.22
K constant	12.5
C constant	98
Sensor resolution	1920×1080
Frame readout time	1/100 (s)
LED frequency	500 to 8000 Hz
Exposure time	1/8000 to 1/1000 (s)

## Appendix A Calculate the Maximum Pixel Value

Presume that $EV_{set}$ is the exposure value to which the camera is set. $EV_{LED}$ and $EV_{BG}$ are the indicated exposure values corresponding to the LED light and background light provided by Equations (22) and (24), respectively.

From Equation (26), the exposure difference is given by:

(A1) $$\begin{array}{c} ED_{max}=\log_{2}\left(2^{ED_{LED}}+2^{EV_{BG}}\right)-EV_{set} \end{array}$$

Then, the maximum pixel value is given by:

(A2) $$\begin{array}{c} PV_{max}=118\times 2^{ED_{max}/\gamma }=118\times {\left(\frac{2^{EV_{LED}}+2^{EV_{BG}}}{2^{EV_{set}}}\right)}^{1/\gamma } \end{array}$$

From Equations (12), (22) and (24), we have:

(A3) $$\begin{array}{c} \left\{\begin{array}{c} 2^{EV_{set}}=\frac{N^{2}}{t}\\ 2^{EV_{LED}}=\frac{L_{v}\times S}{K}\\ 2^{EV_{BG}}=\frac{E_{v}\times S}{C}=\frac{E_{v}\times S}{K}\times \frac{R}{\pi } \end{array}\right. \end{array}$$

Therefore, the maximum pixel value is given by:

(A4) $$\begin{array}{c} PV_{max}=118{\left(\frac{S\times t}{K\times N^{2}}\left(L_{v}+E_{v}\frac{R}{\pi }\right)\right)}^{1/\gamma } \end{array}$$

## Appendix B Calculate the Minimum Pixel Value

In this case, the only light source coming to the sensor is the background ambient light. Presume that $EV_{BG}$ is the indicated exposure values corresponding to the background light provided by Equation (24). The exposure difference is given by:

(B1) $$\begin{array}{c} ED_{min}=EV_{BG}-EV_{set} \end{array}$$

Therefore, the minimum pixel value is given by:

(B2) $$\begin{array}{c} PV_{min}=118\times 2^{ED_{min}/\gamma }=118\times 2^{(EV_{BG}-EV_{set})/\gamma }=118{\left(\frac{S\times t}{K\times N^{2}}\times E_{v}\frac{R}{\pi }\right)}^{1/\gamma } \end{array}$$

## Appendix C Pixel Value of a Row in a Transition Band

Presume that $EV_{set}=\frac{N^{2}}{t}$ is the set exposure value. From Equation (12), the effective exposure value is given by:

(C1) $$\begin{array}{c} EV_{eff}=\log_{2}\frac{N^{2}}{t_{eff}}=EV_{set}+\log_{2}\frac{t}{t_{eff}} \end{array}$$

From Equations (30) and (31), $\frac{t}{t_{eff}}=\frac{h_{t}}{h_{t}-i_{r}}$

Therefore, the effective exposure value is given by:

(C2) $$\begin{array}{c} EV_{eff}=EV_{set}+\log_{2}\frac{h_{t}}{h_{t}-i_{r}} \end{array}$$

Presume $EV_{LED}$ and $EV_{BG}$ are the indicated exposure values corresponding to the LED light and the ambient light. Let $H_{LED}$, $H_{BG}$ and $H_{combine}$ denote the luminous exposure received by the sensor from the LED radiated light, the reflected ambient light and the combined light source, respectively.

From Equations (13) and (15), $H_{LED}$, $H_{BG}$ and $H_{combine}$ are given by:

(C3) $$\begin{array}{c} \left\{\begin{array}{c} H_{LED}=H_{SOS}\times 2^{EV_{LED}-EV_{eff}}\\ H_{BG}=H_{SOS}\times 2^{EV_{BG}-EV_{set}}\\ H_{combine}=H_{LED}+H_{BG} \end{array}\right. \end{array}$$

Then, the exposure difference of the $i_{r}$-th row is given by:

(C4) $$\begin{array}{c} ED_{i_{r}}=\log_{2}\frac{H_{combine}}{H_{SOS}}=\log_{2}\left(2^{EV_{LED}-EV_{eff}}+2^{EV_{BG}-EV_{set}}\right) \end{array}$$

The pixel value of the $i_{r}$-th row is given by:

(C5) $$\begin{array}{c} PV_{i_{r}}=118\times 2^{ED_{i_{r}}/\gamma }=118\times {\left(2^{EV_{LED}-EV_{eff}}+2^{EV_{BG}-EV_{set}}\right)}^{1/\gamma } \end{array}$$

From Equations (C2) and (C5), the pixel value of the $i_{r}$-th row is given by:

(C6) $$\begin{array}{c} PV_{i_{r}}=118{\left(\frac{h_{t}-i_{r}}{h_{t}}2^{EV_{LED}-EV_{set}}+2^{EV_{BG}-EV_{set}}\right)}^{1/\gamma }=118{\left(\frac{S\times t}{K\times N^{2}}\left(L_{v}\frac{h_{t}-i_{r}}{h_{t}}+E_{v}\frac{R}{\pi }\right)\right)}^{1/\gamma } \end{array}$$

## Appendix D Derivative of SINR with Respect to Exposure Time

$\frac{\partial SINR}{\partial t}=\frac{-\left(1/\gamma +1\right)\left({\left(E_{v}\frac{R}{\pi }\right)}^{1/\gamma }-{\left(L_{v}+E_{v}\frac{R}{\pi }\right)}^{1/\gamma }\right)\left({\left(E_{v}\frac{R}{\pi }\right)}^{1/\gamma +1}-{\left(L_{v}+E_{v}\frac{R}{\pi }\right)}^{1/\gamma +1}\right)L_{v}f_{led}}{{\left(\left(1/\gamma +1\right)\left(t\times f_{led}-1\right)L_{v}{\left(E_{v}\frac{R}{\pi }\right)}^{1/\gamma }+t\times f_{led}\left({\left(E_{v}\frac{R}{\pi }\right)}^{1/\gamma +1}-{\left(L_{v}+E_{v}\frac{R}{\pi }\right)}^{1/\gamma +1}\right)\right)}^{2}}$

Since 1/γ>0 and $L_{v}+E_{v}\frac{R}{\pi }>E_{v}\frac{R}{\pi }$, $\frac{\partial SINR}{\partial t}<0$.

## Appendix E Derivative of SINR with Respect to LED Frequency

$\frac{\partial SINR}{\partial f_{led}}=\frac{-\left(1/\gamma +1\right)\left({\left(E_{v}\frac{R}{\pi }\right)}^{1/\gamma }-{\left(L_{v}+E_{v}\frac{R}{\pi }\right)}^{1/\gamma }\right)\left({\left(E_{v}\frac{R}{\pi }\right)}^{1/\gamma +1}-{\left(L_{v}+E_{v}\frac{R}{\pi }\right)}^{1/\gamma +1}\right)L_{v}t}{{\left(\left(1/\gamma +1\right)\left(t\times f_{led}-1\right)L_{v}{\left(E_{v}\frac{R}{\pi }\right)}^{1/\gamma }+t\times f_{led}\left({\left(E_{v}\frac{R}{\pi }\right)}^{1/\gamma +1}-{\left(L_{v}+E_{v}\frac{R}{\pi }\right)}^{1/\gamma +1}\right)\right)}^{2}}$

Since 1/γ>0 and $L_{v}+E_{v}\frac{R}{\pi }>E_{v}\frac{R}{\pi }$, $\frac{\partial SINR}{\partial f_{led}}<0$.

## Appendix F Derivative of SINR with Respect to Ambient Light Illuminance

$\frac{\partial SINR}{\partial E_{v}}=\frac{L_{v}\left(\frac{1}{\gamma }+1\right)\left(t\times f_{led}-1\right)\left({\left({\left(L_{v}+E_{v}\frac{R}{\pi }\right)}^{\frac{1}{\gamma }}-{\left(E_{v}\frac{R}{\pi }\right)}^{\frac{1}{\gamma }}\right)}^{2}t\times f_{led}+{\left(L_{v}+E_{v}\frac{R}{\pi }\right)}^{\frac{1}{\gamma }-1}\frac{1}{\gamma }{L_{v}}^{2}{\left(E_{v}\frac{R}{\pi }\right)}^{\frac{1}{\gamma }-1}\left(1+\frac{1}{\gamma }\left(1-t\times f_{led}\right)\right)\right)}{\frac{\pi }{R}{\left(\left(\frac{1}{\gamma }+1\right)\left(t\times f_{led}-1\right)L_{v}{\left(E_{v}\frac{R}{\pi }\right)}^{\frac{1}{\gamma }}+t\times f_{led}\left({\left(E_{v}\frac{R}{\pi }\right)}^{\frac{1}{\gamma }+1}-{\left(L_{v}+E_{v}\frac{R}{\pi }\right)}^{\frac{1}{\gamma }+1}\right)\right)}^{2}}$

Since $t\times f_{led}=\frac{Exposure\;time}{LED\;duration}<1$, 1/γ>0 and $L_{v}+E_{v}\frac{R}{\pi }>E_{v}\frac{R}{\pi }$, $\frac{\partial SINR}{\partial E_{v}}<0$.

## Appendix G. Derivative of SINR with Respect to LED Luminance

$\frac{\partial SINR}{\partial L_{v}}=\frac{L_{v}\left(\frac{1}{\gamma }+1\right)\left(1-t\cdot f_{led}\right)\left({\left({\left(L_{v}+E_{v}\frac{R}{\pi }\right)}^{\frac{1}{\gamma }}-{\left(E_{v}\frac{R}{\pi }\right)}^{\frac{1}{\gamma }}\right)}^{2}\left(L_{v}+E_{v}\frac{R}{\pi }\right)\left(E_{v}\frac{R}{\pi }\right)t\cdot f_{led}+\frac{1}{\gamma }{L_{v}}^{2}{\left(L_{v}+E_{v}\frac{R}{\pi }\right)}^{\frac{1}{\gamma }}{\left(E_{v}\frac{R}{\pi }\right)}^{\frac{1}{\gamma }}\left(1+\frac{1}{\gamma }\left(1-t\cdot f_{led}\right)\right)\right)}{{\left(\left(\frac{1}{\gamma }+1\right)\left(t\cdot f_{led}-1\right)L_{v}{\left(E_{v}\frac{R}{\pi }\right)}^{\frac{1}{\gamma }}+t\cdot f_{led}\left({\left(E_{v}\frac{R}{\pi }\right)}^{\frac{1}{\gamma }+1}-{\left(L_{v}+E_{v}\frac{R}{\pi }\right)}^{\frac{1}{\gamma }+1}\right)\right)}^{2}}$

When $E_{v}=0$, $\frac{\partial SINR}{\partial L_{v}}=0$. When $E_{v}>0$, since $t\times f_{led}<1$, 1/γ>0 and $L_{v}+E_{v}\frac{R}{\pi }>E_{v}\frac{R}{\pi }$, $\frac{\partial SINR}{\partial L_{v}}>0$.
